# Experimental study on treatment of acetabular anterior column fractures: applyment of a minimally invasive percutaneous lag screw guide apparatus

**DOI:** 10.1186/s12891-015-0846-1

**Published:** 2016-01-15

**Authors:** Li-hai Zhang, Li-cheng Zhang, Qing-hua Si, Yuan Gao, Xiu-yun Su, Zhe Zhao, Pei-fu Tang

**Affiliations:** Department of Orthopedics, Chinese PLA General Hospital, No.28 Fuxing Road, Haidian District, Beijing, 100853 People’s Republic of China; Department of Orthopedics, Affiliated Hospital of the Academy of Military Medical Sciences, No.8 Dongdajie Road, Beijing, 100071 People’s Republic of China; Department of Orthopedics, Beijing Tsinghua Chang Gung Hospital, No.1 Block Tiantongyuan North, Beijing, 102218 People’s Republic of China

**Keywords:** Acetabula anterior column, Fracture, Minimally invasive, Percutaneous, Lag screw, Guide apparatus

## Abstract

**Background:**

The aim of this study was to design a new minimally invasive percutaneous lag screw guide apparatus and to verify its adjuvant treatment of acetabular anterior column fracture on pelvis specimens.

**Methods:**

This guide apparatus was self-developed based on the principles of “two points form a line” and “Rectangle”. Using C-arm fluoroscopy, this guide apparatus was used to conduct minimally invasive percutaneous lag screw internal fixation of acetabular anterior column fractures. Ten hollow lag screws were placed into 5 pelvis specimens.

**Result:**

Result showed no sign of any screws puncturing the cortex or entering into the hip joint on radiological assessment. The cross-section reconstructed vertical distance to the screw, on the cross-section acetabular notch and the cross-section of the screw where the distance of between the screw and the iliopectineal line’s arc roof was at its shortest, indicate that at all points (T, R-r) under the line with an inclination of 1 (namely T = R-r) the screw is within the cortex and does not puncture the acetabula anterior column or enter into the hip joint.

**Conclusions:**

We may conclude that this self-developed guide apparatus solves the screw precision problem during the treatment of acetabular anterior column fractures through a minimally invasive percutaneous lag screw.

## Background

Pelvic fractures are mainly caused by high-energy injuries and are usually followed by multiple injuries. The traditional open reduction and internal fixation (ORIF) treatment will aggravate the injury; therefore, Roberts [1] claimed that in the early period of the injury, ORIF treatment is not indicated and that a minimally invasive percutaneous technique should be adopted. In recent years, drawing support from the development of imaging and navigation technology, the minimally invasive percutaneous lag screw technique has become one of the most commonly used treatment methods because of its advantages of causing minimal injury, decreased surgical time, less complication and so on.

Minimally invasive percutaneous lag screw is a stable and reliable technology that has slight injury [2]. For traditional treatment of displacement or slightly displacement acetabula anterior column, minimally invasive percutaneous the lag screw technique is generally divided into anterograde and retrograde techniques. A vast majority of domestic and foreign scholars have studied the entry point and puncture angle of the anterograde and retrograde techniques [3–9].

For the traditional minimally invasive percutaneous lag screw technique, the position of entry point is always determined first, followed by the direction. The puncture angle has strong subjectivity, because there is no supporting point in the acetabular and no correct direction when the guiding needle drilled during the operation. During the drilling, X-ray fluoroscopy is repeatedly used to determine the direction and to obtain satisfactory results. In addition, the need for readjustment of the needle depends on the operator’s subjective feeling, which obviously prolongs the time of the operation and increases the X-ray exposure time for the physician and the patient. Carmack [10] showed that although fluoroscopy is repeatedly used, the screw steel may deviate from the correct position.

Based on the principle of “two points form a line”, the position and direction can be determined by the screw’s entry and exit points. To self-develop minimally invasive percutaneous lag screw guides the apparatus has to ensure that the guiding needle or screw gets through the entry and exit point and enter the anterior column cortex, to reduce the complication of screw puncturing out, simplify the operation and reduce the radiation during the operation.

## Methods

### Materials

This in vitro study was approved by the Ethics Committee of Chinese PLA General Hospital. Due to the fact that the cadaver pelvises used in this study were provided by Anatomy Lab of Chinese Aerospace General Hospital, no informed consent was needed. For this study we used 5 pelvises, self-made pelvis upper and lower pads, a C-arm X-ray machine (SIEMENS ARCADIS ORBIC), minimally invasive percutaneous lag screw guiding apparatus, and protractor.

### The design principle of minimally invasive percutaneous lag screw guiding apparatus

**Fig. 1:**
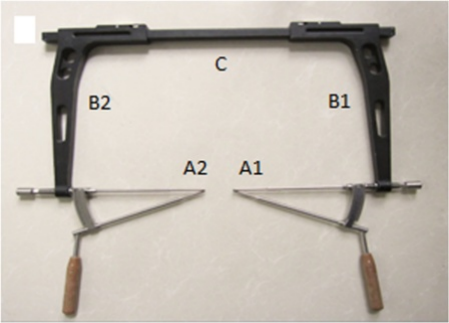
The design of minimally invasive percutaneous lag screw guiding apparatus

The principle of “Two Points Form a Line” helps determine the position of the two points, the entry and exit points. Based on the structure of the rectangle, which shows that the length of the corresponding two sides are equal, the minimally invasive percutaneous lag screw guide apparatus is developed and is expected to solve the difficult problem that the hollow screw cannot be placed successful in the acetabula anterior column (Fig. 1).

### The usage and surgical indication of minimally invasive pecutaneous lag screw guide apparatus

**Fig. 2:**
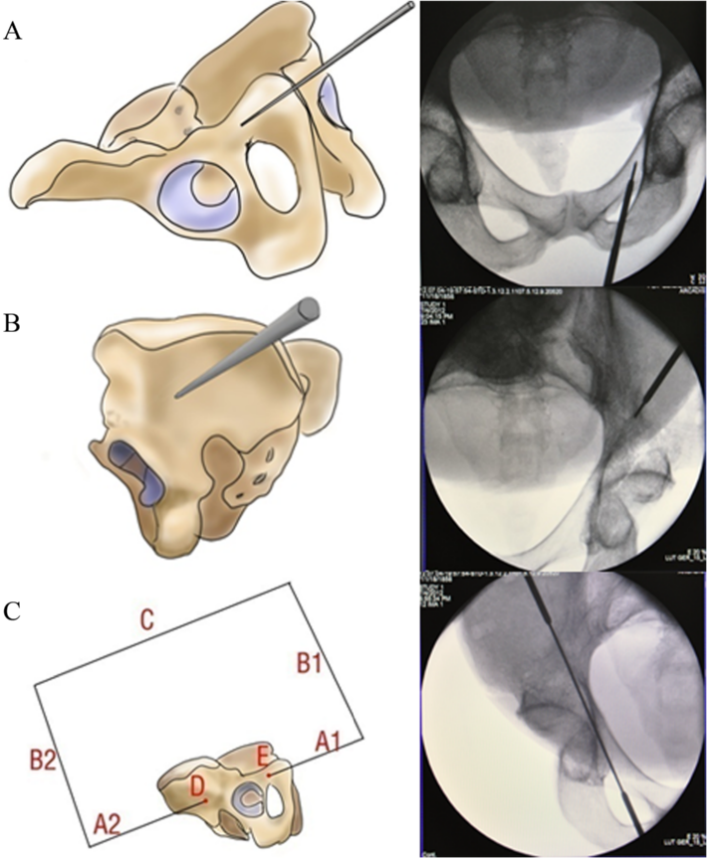
**a** Locater (A1) is fixed to the obturator crest entry point of acetabular anterior column **b** Locater (A2) is fixed to the posterior column of ilium outside entry point. **c** The X-ray of the guide apparatus form the rectangle structure and the guiding needle enters the opposite locater (the guiding needle enters the opposite locater by passing through the obturator crest entry point (E) and the posterior column of the ilium outside the entry point (D))

This apparatus has two important parts, including two locators (A1 and A2), two side connection bars (B1 and B2) and one square connection bar (C), which ensure that the entire apparatus form a rectangle and fulfill the principle of “Two Points Form a Line”. (See Fig. 1) C arm navigation should be applied during the surgery. First, the entry position (E in Fig. 2) is determined by a locater (A1 in Figs. 1 and 2) and the exit position (D in Fig. 2) by another locater (A2 in Figs. 1 and 2). Then, B1 and B2 are connected by a square connection bar (C in Figs. 1 and 2). Lastly, an electric drill is used to drill from locater (A1) to locater (A2) or from locater (A2) to locater (A1). Finally, the guiding needle moves through entry and exit points (D and E in Fig. 2) successfully and the hollow screw is secured. (See Fig. 2) The surgical indication of this apparatus includes simple fracture of the anterior column of the acetabulum with minimal or no displacement after miniinvasive or closed anatomical reduction and the transverse fracture of the acetabulum with minimal or no displacement.

### Operation mimesis in vitro

**Fig. 3:**
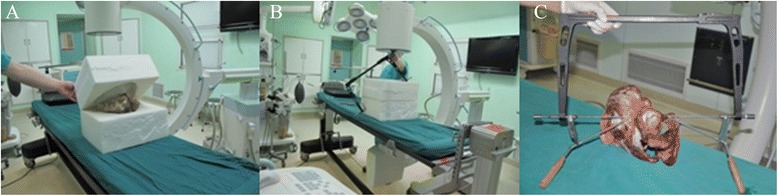
**a** The pelvis is completely wrapped up with the self-made pelvis pad. **b** Form a rectangle structure. **c** The guiding needle passes successfully through the opposite locater

Place the pelvis specimens in the self-made pelvis lower pad, keep the plane of the pelvis at 60° to the horizontal plane and then cover the self-made pelvic upper pad (Fig. 3a). Let the pelvic completely be wrapped up by the pelvis pad to simulate a patient who is lying flat on the operation table. The C-arm is placed on the contralateral side. Conventional views obtained are frontal, inlet and outlet views of the pelvis, the obturator oblique view and iliac oblique view. Following the steps mentioned above, the guiding needle is successfully placed through the locater tube of the posterior column of the ilium (Fig. 3). Then, the guide apparatus is used and the hollow lag screw is attached along with the guiding needle.

### Evaluation Index

#### Visual observation

When the operation was finished, the five pelvises were visually observed to find out whether the hollow lag screw punctured the cortex or entered into the hip joint

#### X-ray examination

When the operation was finished, the five pelvises were placed under the C-arm to obtain views from the front and back, as well as inlet and outlet view of pelvis to confirm whether the guiding needle is in the acetabular anterior column.

#### CT Scan and analysis

**Fig. 4:**
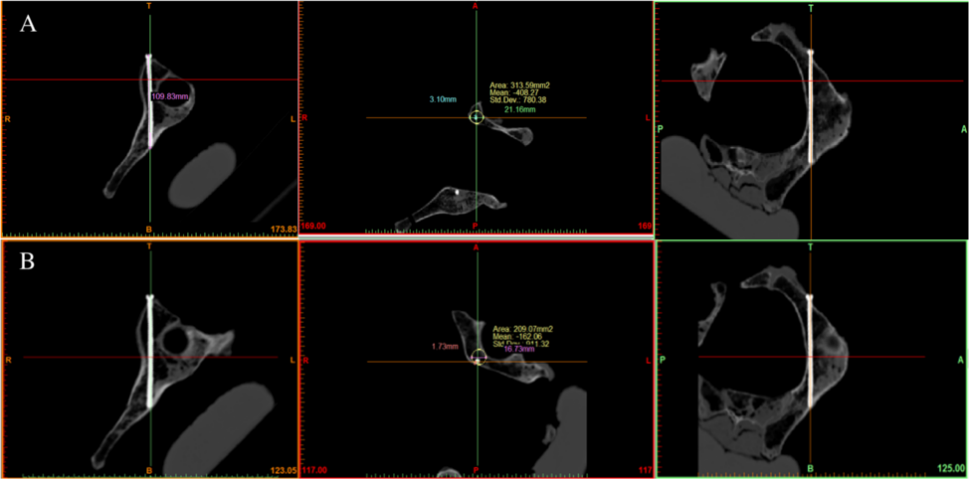
Using the reslice function of the Mimics software, the cross-section vertical to the screw is reconstructed. R refers to the radius of the containable biggest circle. r refers to the radius of the hollow screw. T refers to the distance between the center of the containable biggest circle and the screw center. **a** shows the cross-section reconstructed on the acetabular notch. **b** shows the cross-section when the distance of screw and iliopectineal line arc roof is the minimum

After the experiment, CT scan and 3-D reconstructions were obtained for the five pelvises to confirm the position of the screws. The CT data was entered into Mimics by .docm Format and a cross-section vertical to the screw by reslice function was obtained along the Screw (Fig. 4).

The authors reconstructed the cross-section of acetabular notch (Fig. 4a) and the two cross-sections of the screw at which the distance of the screw and iliopectineal line’s arc roof is at its shortest (Fig. 4b). Within the two cross-sections, the radius of the biggest circle containing R (mm) and the distance of the circle center to the screw center T (mm) are measured. Here we used a 5.0-mm diameter hollow screw (the radius is 2.5 mm).

### Make scatter diagram to describe distribution

The scatter diagram was obtained with the measured data (R-r) as the transverse axis and T as the vertical y-axis to describe the distribution. When points (T, r) were under the line with an inclination of 1 (namely T = r-r), the screw was suggested to be within the cortex and not puncturing the acetabula anterior column or the hip joint.

## Results

After the minimally invasive percutaneous lag screw fixation operation on acetabular anterior column fractures were performed, 10 hollow lag screws were placed into five pelvises using the minimally invasive percutaneous lag screw guide apparatus. All the screws were placed in one trail.

### Visual observation

Visually, we observed the five pelvises and no hollow lag screw could be seen puncturing the cortex or entering the hip joint.

### X- ray examination

**Fig. 5:**
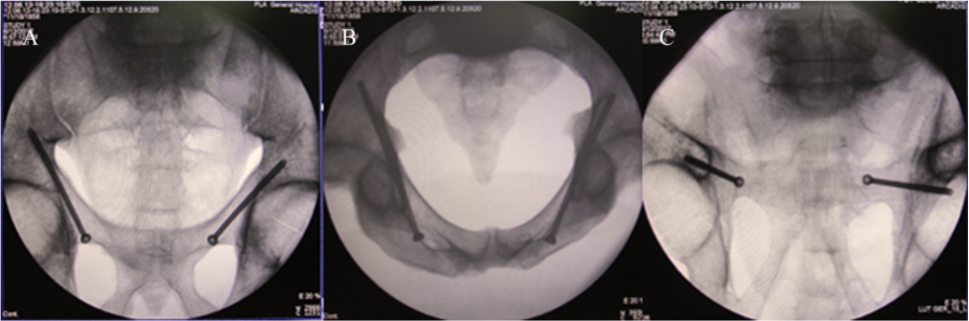
**a** Front and back view of pelvis; **b** Inlet view of pelvis; **c** Outlet view of pelvis

When the operation was completed, the five pelvises were examined under the C-arm to obtain views from the front and back, as well as inlet and outlet views of the pelvis. The results are presented in Fig. 5.

### CT scan and 3-D reconstruction

**Fig. 6:**
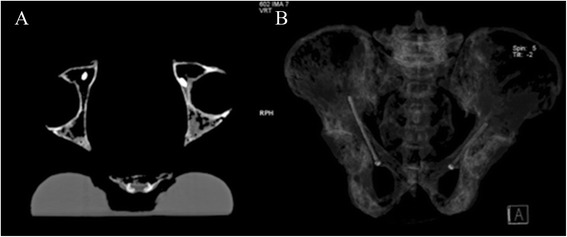
**a** Pelvis CT scan. **b** Pelvis CT 3-D reconstruction

After the experiment, CT scan and 3-D reconstructions were obtained for the five pelvises to confirm the position of the screws further (see Fig. 6).

### Make the scatter diagram

**Fig. 7:**
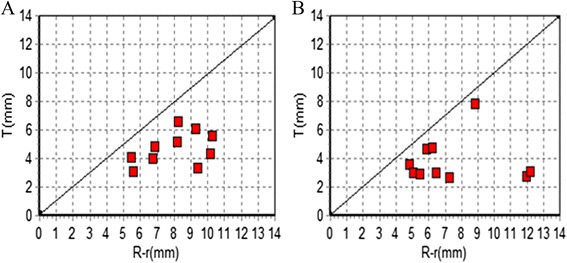
**a** shows the cross-section of the acetabular notch, which presents the relationship between points (T, R-r) and the line, which has an inclination of 1 (namely T = r-r). **b** shows the minimum distance between the screw and the iliopectineal line’s arc roof, which presents the relationship between points (T, R-r) and the line with an inclination of 1 (namely T = r-r)

In Fig. 7, (R-r) represents the transverse axis and T represents the vertical y-axis. On the cross-section of the acetabular notch and the two cross-section of the screw in which the distance of the screw and iliopectineal line’s arc roof is at its shortest, when all points (T, r) are under the line which is at an inclination of 1 (namely T = r-r), the screw is suggested to be within the cortex and not puncturing the acetabula anterior column or entering the hip joint.

## Discussions

Acetabula anterior column fractures are caused by high-energy injury. The traditional method is to treat with open reduction and internal fixation. However, this approach has disadvantages such as significant tissue injury, blood lost and slower recovery. The wound is easily infected and may result in injury to blood vessels and nerves and heterotopic ossification [11–14]. Therefore, the traditional method has limitations in clinical application. The concept of using percutaneous screw fixation to treat acetabula anterior column has been gradually developed. Compares with the traditional open reduction and plate internal fixation, this technique has the following advantages decreased injury, less blood loss, and shorter operative time. There is no need to strip the soft tissue around the fracture site, which is beneficial to the recovery of the bone, and it has a reliable stability, which is why the patients can exercise sooner. In summary, less complication is beneficial to the recovery. Hollow screws provide sound fixation on mechanics, and the stability of bio-mechanic is equal to the stability of plate [15].

However, due to the high technical requirement, it depends to a large extent on the operator’s subjective feeling and lacks objective evidences. The guiding needle will definitely deviate from the correct position and puncture the cortex and enter into the pelvis cavity or the hip joint causing injury to blood vessels and nerves. Therefore, its clinical application is limited [8, 16]. The traditional minimally invasive percutaneous lag screw technique needs to rely on repeated X-ray fluoroscopy to obtain satisfactory surgical results, which prolongs the surgical time and increases the X-ray exposure time for the physician and patient. Although X-ray fluoroscopy is repeatedly used, the screw steel deviates from the correct position [10].

By introducing the principle of “two points form a line”, the guide apparatus dramatically simplifies and improves the precision of the screw placement. The task group shaped the device to a rectangle. During the operation, a screw can be accurately placed under the guidance of the guiding needle, whose direction has been determined by two points on the four sides of the rectangle as the entry and exit points. In other words, as long as the two points on the guide apparatus are determined, precise screw placement can be achieved.

Another obvious advantage of the guide apparatus is that when the guide apparatus is used to place anterior column hollow screws, there is no need to shoot X-ray images, such as the traditional front, inlet and outlet views and to repeatedly obtain images to confirm the position and direction of the guiding needle during the operation. The guiding apparatus does not need repeated fluoroscopy instead the obturator oblique and the iliac oblique views are needed to determine the position of the two points. The group’s experiment reduced the times of radioscopy and shortened the operation time by using the minimally invasive percutaneous lag screw guide apparatus.

The guide apparatus needs further verification with follow up cadaver experiments and clinical application.

## Conclusions

In conclusion, the proposed minimally invasive pecutaneous lag screw guide apparatus should be used after miniinvasive or closely anatomical reduction of fractures, which include manual reduction, traction and locations with several K-wires under intro-operative fluoroscopy,and based on comprehensive knowledge of the pelvic anatomy and fractures. According to the principle of “two points form a line” and the shape of the Rectangle, the self-developed minimally invasive percutaneous lag screw guide apparatus solves the screw precision problem for the treatment of acetabular anterior column fracture with minimally invasive percutaneous lag screw and is a feasible and effective apparatus for its treatment.
